# Role of Silicon Counteracting Cadmium Toxicity in Alfalfa (*Medicago sativa* L.)

**DOI:** 10.3389/fpls.2016.01117

**Published:** 2016-07-27

**Authors:** Ahmad H. Kabir, Mohammad M. Hossain, Most A. Khatun, Abul Mandal, Syed A. Haider

**Affiliations:** ^1^Plant and Crop Physiology Laboratory, Department of Botany, University of RajshahiRajshahi, Bangladesh; ^2^System Biology Research Center, School of Bioscience, University of SkövdeSkövde, Sweden

**Keywords:** Alfalfa, silicon, Fe regulation, antioxidant activities, Cd stress

## Abstract

Cadmium (Cd) is one of the most phytotoxic elements causing an agricultural problem and human health hazards. This work investigates whether and how silicon (Si) ameliorates Cd toxicity in Alfalfa. The addition of Si in Cd-stressed plants caused significant improvement in morpho-physiological features as well as total protein and membrane stability, indicating that Si does have critical roles in Cd detoxification in Alfalfa. Furthermore, Si supplementation in Cd-stressed plants showed a significant decrease in Cd and Fe concentrations in both roots and shoots compared with Cd-stressed plants, revealing that Si-mediated tolerance to Cd stress is associated with Cd inhibition in Alfalfa. Results also showed no significant changes in the expression of two metal chelators [*MsPCS1* (phytochelatin synthase) and *MsMT*2 (metallothionein)] and PC (phytochelatin) accumulation, indicating that there may be no metal sequestration or change in metal sequestration following Si application under Cd stress in Alfalfa. We further performed a targeted study on the effect of Si on Fe uptake mechanisms. We observed the consistent reduction in Fe reductase activity, expression of Fe-related genes [*MsIRT1* (Fe transporter), *MsNramp1* (metal transporter) and *OsFRO1* (ferric chelate reductase] and Fe chelators (citrate and malate) by Si application to Cd stress in roots of Alfalfa. These results support that limiting Fe uptake through the down-regulation of Fe acquisition mechanisms confers Si-mediated alleviation of Cd toxicity in Alfalfa. Finally, an increase of catalase, ascorbate peroxidase, and superoxide dismutase activities along with elevated methionine and proline subjected to Si application might play roles, at least in part, to reduce H_2_O_2_ and to provide antioxidant defense against Cd stress in Alfalfa. The study shows evidence of the effect of Si on alleviating Cd toxicity in Alfalfa and can be further extended for phytoremediation of Cd toxicity in plants.

## Introduction

Cadmium (Cd) is a toxic heavy metal affecting plant yield and environment (Järup and Åkesson, 2009; Naeem et al., 2015). It exhibits varied degrees of phytotoxicity and may replace essential metals or cofactors at enzyme active site causing an imbalance in cellular redox status (Hattab et al., 2014). Furthermore, Cd at toxic level displaces protein structure and disrupts membrane integrity (Rascio and Navarri-Izzo, 2011). Cd is naturally high in agricultural soils due to anthropogenic release and is frequently accumulated in plants. Alfalfa (*Medicago* *sativa* L.) is an important forage crop and can accumulate heavy metals, such as Cd, Ni, Cu, Zn, Vn (Peralta-Videa et al., 2002; Yang et al., 2011; Carrasco-Gil et al., 2012). Alfalfa is a source of biological nitrogen fixation, bio-fuel and animal feed. Therefore, reducing Cd bioaccumulation in Alfalfa deserves attention.

Silicon (Si) is an abundant element in the Earth’s crust and plays a role in heavy metal alleviation in plants by different mechanisms (Ma et al., 2008; Greger et al., 2016). One mechanism of Cd detoxification is the reduction of Cd uptake into the plant (Treder and Cieslinski, 2005). Also, Si reduces the translocation of Cd from roots to shoots and thus, prevents the adverse effect of Cd on photosynthetic machinery and grains (Greger and Landberg, 2008; Zhang et al., 2008). However, Cd in high concentration is also trapped in roots through vacuolar sequestrations (Greger et al., 2016), leading to decreased Cd translocation in aerial parts of the plants (Lindberg et al., 2007; Liu et al., 2013). Phytochelatins (PCs) and metallothioneins (MTs) may bind to Cd before transporting the complexes into the vacuole or out of the cell by ATP-binding cassette transporters in few plants (Cobbett, 2000; Jasinski et al., 2003). PCs are formed from glutathione by the induction of *PCS1* gene (Semane et al., 2007). Further, MTs are involved in detoxifying cytosolic environment of the cell from Cd toxicity (Cobbett and Goldsbrough, 2002; DalCorso et al., 2010).

The Cd is taken up into the cell via carriers, such as low-affinity cation transporters and Fe-regulated transporters in plants (Lindberg et al., 2004; Takahashi et al., 2011). Among the Fe transporters, *IRTs* and NRAMPs have been reported to take up Cd as well as Fe (Curie et al., 2000; Nakanishi et al., 2006). *IRT1* is essential for root Fe uptake in response to Fe deficiency but it also accepts Cd as a substrate and is involved in the root-to-shoot transport of Cd (Rogers et al., 2000). In a transgenic study, elimination of *NRAMP5* transporter reduces Cd uptake in rice (Ishikawa et al., 2012).). Additionally, the ferric chelate reductase (*FRO*) gene may perform key functions in Fe acquisition in plants (Ling et al., 2002). Barcelo et al. (1993) reported the inhibition of Fe translocation when bean plants were exposed to chromium (Cr) in nutrient solutions. Also, Cr affects Fe uptake in dicots either by inhibiting the reduction of Fe(III) to Fe(II) or by competing with Fe(II) at the site of absorption (Shanker et al., 2005). In addition, *IRT1* is induced in response to Fe-deficiency and is capable of transporting minerals and heavy metals (Vert et al., 2002). Further, organic acids such as citrate and malate are major chelators in both Strategy I and II plants, which bind Fe at the site of uptake and facilitate long-distance transport in plants (Abadia et al., 2002; Kabir et al., 2012). Therefore, regulation of Fe uptake has yet to be characterized in detail under Cd stress in plants.

Abiotic stresses generate excessive reactive oxygen species (ROS) which are relatively reactive compared with O_2_ and may lead to the unspecific oxidation of proteins and membrane lipids or may cause DNA injury (Bolwell et al., 2002; Schützendübel and Polle, 2002). In plants, the control of oxidative burst is achieved by antioxidative systems. These defense systems are composed of enzymatic scavengers, such as catalase (CAT), peroxidase (POD), superoxide dismutase (SOD), glutathione reductase (GR), ascorbate peroxidase (APX) and antioxidant metabolites. Among these enzymes, SOD is the front line of defense against ROS, dismutating reactive O_2_ to an oxygen molecule and H_2_O_2_ (Irfan et al., 2014). Further, an increase in the cellular ROS is subsequently converted to H_2_O_2_ (Hung et al., 2005). H_2_O_2_ can be directly decomposed through CAT or H_2_O_2_ can also be removed via recurrent oxidation–reduction reactions promoted by glutathione (Schützendübel and Polle, 2002). Further, APX enzymes use ascorbate as an electron donor to catalyze the conversion of H_2_O_2_ into H_2_O (Caverzan et al., 2012). In addition, some metabolites (i.e., glutathione, cysteine, glycine, methionine, proline) play critical roles to alleviate the damage induced by metal stress (Sharma and Dietz, 2006; Kumar et al., 2014; Kabir, 2016). Glutathione and proline may also function as sources of reduced-S and N under stress (Anjum et al., 2014).

Although the role of Si in Cd alleviation is reported in popular crop species, the physiological and molecular basis of Si-mediated Cd tolerance in Alfalfa is still unknown. Moreover, exact mechanisms behind the beneficial role of Si might be species dependent, which should be clearly understood for the improvement of Alfalfa. Therefore, our aim was to investigate whether, how and where Si alleviates Cd toxicity in Alfalfa by morpho-physiological investigations. Further, a combination of molecular, enzymatic and metabolomics analysis was performed in roots to elucidate the mechanisms associated with Si-mediated mitigation of Cd toxicity in Alfalfa.

## Materials and Methods

### Plant Cultivation

We used Alfalfa var. Longdong (popularly known as drought tolerant) since this cultivar is efficient in utilizing Si. Firstly, seeds were disinfected by superficial treatment with 95% (v/v) ethanol for 10 min before germination. Only uniform seedlings were transferred to the solution culture (Hoagland and Arnon, 1950) containing the following nutrient concentrations (μM): KNO_3_ (16000), Ca(NO_3_)_2_.4H_2_O (6000), NH_4_H_2_PO_4_ (4000), MgSO_4_.7H_2_O (2000), KCl (50), H_3_BO_3_ (25), Fe-EDTA (25), MnSO_4_. 4H_2_O (2), ZnSO_4_ (2), Na_2_MoO_4_.2H_2_O (0.5), and CuSO_4_.5H_2_O (0.5). The nutrient medium was treated with 0 or 1 mM CdCl_2_ and 0 or 1 mM K_2_SiO_3_ as previously described (Greger et al., 2016). The pH of the nutrient media was adjusted to 6.0 by using NaOH or HCl. The plants were harvested for analysis after treatment for 7 days. The nutrient solutions were continuously aerated and the growth chamber was strictly maintained under 10 h light and 14 h dark (550–560 μmol s^-1^ per μA).

### Measurement of Morphological Features and Chlorophyll Concentration

Morphological growth parameters, such as root length, root dry weight, shoot height, shoot dry weight were measured on 1-week-old plants. The roots and leaves of the plants were separated manually and then dried in an oven at 80°C for 2 days before taking the dry weight. Total chlorophyll concentration (a and b) of leaves was determined as previously described (Lichtenthaler and Wellburn, 1985). Firstly, the leaf was weighed and placed in 95% acetone in a 5 mL falcon tube. The leaf sample was then ground with mortar-pestle and centrifuged at 12000 *g* for 10 min. The absorbance of the separated supernatant was read at 662 nm (chlorophyll a) and 646 nm (chlorophyll b) on a spectrophotometer (UV-1650PC, Shimadzu). The amount of these pigments was calculated based on the formula given by Lichtenthaler and Wellburn (1985).

### Determination of Cd and Fe by AAS (Atomic Absorption Spectroscopy)

Harvested roots and shoots were washed with CaSO_4_ and deionized water before drying in an oven at 80°C for 3 days. The dried samples were then digested in 3 mL HNO_3_ before heating at 75°C for 10 min. Further, 1 mL of H_2_O_2_ was added to each vessel through the ventilation hole and then heated at 109°C for 15 min. The samples were then analyzed for Cd and Fe concentrations by Flame atomic absorption spectroscopy (AAS) outfitted with the ASC-6100 autosampler and air-acetylene atomization gas mixture system (Model No. AA-6800, Shimadzu). Standard solutions of Cd and Fe were prepared separately from their respective concentrations of stock solutions to cover the optimum absorbance ranges for the standard calibration curve.

### Determination of Total Soluble Proteins

Total soluble proteins were extracted from roots and shoots as previously described (Guy et al., 1992) with some modifications. The samples were homogenized with a chilled mortar and pestle in buffer containing ice-cold 50 mM Tris-HCl, pH 7.5; 2 mM EDTA and 0.04% (v/v) 2-mercaptoethanol. The homogenate was centrifuged at 12000 *g* for 30 min at room temperature and the supernatant was then transferred (100 μl) to glass cuvette containing 1 ml of Coomassie Brilliant Blue G 250 (CBB). The absorbance was recorded at 595 nm and the concentration of total soluble protein was calculated using the calibration curve of Bovine serum albumin (BSA).

### Measurement of Electrolyte Leakage

Electrolyte leakage (EL) was measured in roots and shoots using an electrical conductivity meter (Lutts et al., 1996). Seedling samples were washed with deionized water to remove surface contamination, weighed and placed in individual vials containing 20 mL of deionized water. Samples were then incubated at 25°C on a shaker (100 rpm) for 2 h. The electrical conductivity of the solution was then read after incubation.

### Determination of Si Concentration

Roots and leaves were washed with CaSO_4_ and deionized water before grinding in mortar-pestle. The samples were then centrifuged at 12000 *g* for 5 min and supernatant was transferred to 10% ammonium molybdate. Afterward, 10% oxalic acid was added to the sample mixture to form a silico-molybdate complex. Further, 0.5% ascorbic acid was added to the mixture and kept for 20 min at room temperature until blue color is formed. The absorbance of the reaction mixture was then measured at 660 nm in a UV spectrophotometer.

### Analysis of Plant Metabolites by HPLC (High-Performance Liquid Chromatography)

Plant metabolites were analyzed in roots harvested from 1-week-old plants by HPLC (Binary Gradient HPLC System, Waters Corporation, Milford, MA, USA) with Empower2^TM^ software as previously described with some modifications (Kabir et al., 2015). The HPLC systems comprised a Waters 515 HPLC pump and Waters In-line degasser AF. For compound separation, a C18 reverse phase-HPLC column (particle size: 5 μm, pore size: 300 A, pH Range: 1.5–10, Dimension: 250 mm × 10 mm) was attached. In mobile phase, buffer A (water and 0.1% TFA) and buffer B (80% acetonitrile and 0.1% TFA) were used at the gradient of: 1–24 min 100% A, 25–34 min 100% B and 35–40 min 100% A. Standards and sample extracts were diluted (100×) and subsequently filtered using 0.22 μm Minisart Syringe Filters (Sartorius Stedim Biotech, Germany) before injection. PC were then detected with a Waters 2489 dual absorbance detector (Waters Corporation, Milford, MA, USA) at 280 and 360 nm. For PC analysis, the peak was detected with *Thlaspi arvensis* in comparison with the retention times. GSH-equivalents of each PC were further used for PC quantification (Lindberg et al., 2007).

### Fe Chelate Reductase Activity

Fe (III)-reductase activity was measured in roots and shoots using ferrozine [3-(2-pyridyl)-5, 6-diphenyl-1,2, 4-triazine sulfonate] as previously described (Kabir et al., 2015). Washed root tips were cut and placed in a beaker filled with ice water. About 0.1 g of tissue was soaked in 1 mM EDTA for 5 min to eliminate apoplastic Fe and washed three times with distilled water to reduce excess EDTA. The roots were then transferred to 50 mL assay solution containing 0.10 mM MES-NaOH (pH 5.5), 0.5 mM CaSO_4_, 100 mM Fe(III) EDTA, and 300 mM ferrozine. Samples and control tubes were incubated for 1 h in a shaking water bath at 14,000 rpm at 23°C in the dark. After incubation, a 1 mL aliquot from each tube was transferred into a cuvette and the absorbance was measured with a spectrophotometer (UV-1650PC, Shimadzu) at 562 nm wavelength. Reduced Fe [Fe(II)] was calculated with the use of an extinction coefficient of 25,200 M^-1^ cm^-1^.

### RNA Isolation and Quantitative Real-Time PCR

Expression analysis of *Actin, MsPCS1, MsMT2, MsIRT1, MsNRAMP1, and MsFRO1* was performed by quantitative qRT-PCR (reverse transcription PCR) in roots of 1-week-old plants. Briefly, tissues (50–100 mg) were ground with a mortar and pestle to a fine powder in liquid nitrogen. Afterward, total RNA was isolated according to the protocol supplied by SV Total RNA Isolation System (cat. no. Z3100), Promega Corporation, USA. The integrity of isolated RNA was then checked by denaturing agarose gel electrophoresis and quantified by NanoDrop 2000 UV-Vis Spectrophotometer. The first-strand cDNA was then synthesized by using GoScript^TM^ Reverse Transcription System (Cat no. A5001), Promega Corporation, United States. Before real-time analysis, the cDNA samples were treated with RNase to eliminate RNA contamination. Real-time PCR was performed in triplicate using the Eco^TM^ real-time PCR system (Illumina, United States) using GoTaq^®^ qPCR Master Mix (Promega, USA) and gene specific primers designed from the available gene sequences of *Medicago sativa* or *Medicago truncatula* (Supplementary Table S1). All primers were used to perform BLASTN searches with *Arabidopsis* genome database to confirm that they would specifically amplify the gene of interest. Expression data set was normalized with *Actin* as an internal control (Eco Software v4.0.7.0). The real-time PCR program used was as follows: 3 min at 95°C, 40 cycles of 30 s at 94°C, 15 s at 58°C and 30 s at 72°C.

### Enzymatic Analysis

Catalase, APX, POD, SOD and GR enzymes were extracted in roots of on 1-week-old plants as previously described with slight modifications (Goud and Kachole, 2012). Briefly, 100 mg of root tissue was ground in 5 mL phosphate buffer (100 mM) and centrifuged at 12000 *g* for 10 min to separate supernatant. For CAT analysis, the reaction mixture (2 mL) contained 100 mM potassium phosphate buffer (pH 7.0), 6% H_2_O_2_ and root extract. After adding the root extract, the decrease in absorbance was recorded at 240 nm (extinction coefficient of 0.036 mM^-1^ cm^-1^) using a UV spectrophotometer at 30 s intervals up to 1 min. APX was analyzed in a reaction mixture containing 50 mM potassium phosphate buffer (pH 7.0), 0.5 mM ascorbic acid, 0.1 mM EDTA, 0.1 mM H_2_O_2_, and 0.1 ml extract. The specific activity of the enzyme was calculated using extinction coefficient of 2.8 mM^-1^ cm^-1^ based on absorbance at 290 nm (Almeselmani et al., 2006). In case of POD, the reaction mixture (2 mL) contained 100 mM potassium phosphate buffer (pH 6.5), 0.05 M pyrogallol, 200 mM H_2_O_2_ and 100 μl root extract. Similarly, the changes in absorbance were measured at 430 nm (extinction coefficient 12 mM^-1^ cm^-1^) in a spectrophotometer from 30 s up to 1.5 min. Furthermore, SOD assay mixture contained 50 mM sodium carbonate/bicarbonate buffer (pH 9.8), 0.1 mM EDTA, 0.6 mM epinephrine and enzyme (Sun and Zigman, 1978). Once epinephrine is added, adrenochrome formation was then monitored for 4 min at 475 nm in a UV-Vis spectrophotometer. Lastly, 100 μl of root extract was added to the assay mixture containing 1 mL of 0.2 M phosphate buffer (pH 7.0) with 1 mM EDTA, 20 mM oxidized glutathione (GSSG) and 2 mM NADPH for GR analysis. Oxidation of NADPH by GR was then recorded at 340 nm (Halliwell and Foyer, 1978). The hydrogen peroxide (H_2_O_2_) content was determined in roots and shoots as previously described (Alexieva et al., 2001). Briefly, tissues were crushed in 0.1% trichloroacetic acid (TCA) and centrifuged at 10, 000 *g* for 15 min. The supernatant was kept in dark for 1 h before mixing with phosphate buffer (10 mM, pH 7.0) and potassium iodide (M). The absorbance of the mixture was recorded at 390 nm.

### Statistical Analysis

All experiments were performed in completely randomized block design and having at least three independent replications for each sample. Statistical significance for each group of data was set at *P* ≤ 0.05 by ANOVA one-way followed by Duncan’s Multiple Range Test (DMRT) using SPSS Statistics 20 Software. Further, the graphical presentation was prepared using GraphPad Prism 6.

## Results

### Morpho-Physiological Features

**FIGURE 1:**
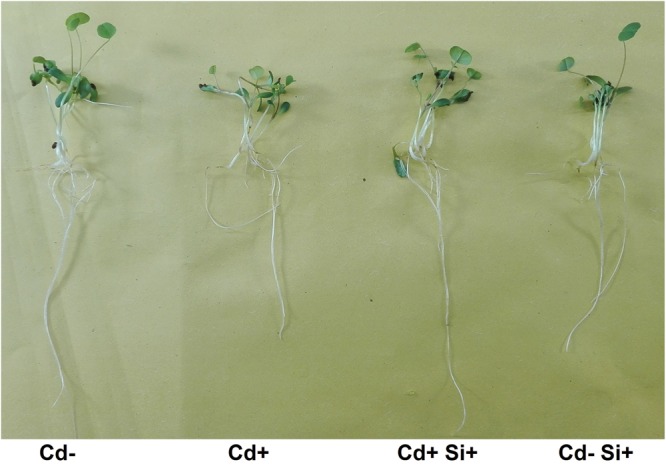
**Phenotypes of 7-days-old Alfalfa plants grown under different growth conditions of Cd and Si**.

Root length, root dry weight, shoot height, shoot dry weight, and total chlorophyll concentration (*a* and *b*) significantly (*P* < 0.05) decreased due to Cd stress compared with non-treated controls (**Table 1**; **Figure 1**). However, these parameters significantly increased due to Si application in combination with Cd treatment compared with Cd-stressed plants. Si supplementation in non-stressed plants caused an adverse effect on root length and shoot dry weight compared to that observed for Cd-stressed plants but showed similar root dry weight and chlorophyll concentration to that of control plants (**Table 1**). However, results showed a non-significant change in morphological features between untreated controls and Si-treated Cd-stressed plants (**Table 1**).

**Table 1:** Morpho-physiological features of Alfalfa after cultivation for 7 days in nutrient medium with or without Cd and Si.

Treatments	Root length (cm)	Root dry weight (mg)	Shoot height (cm)	Shoot dry weight (mg)	Total chl *a* and *b* (mg^-1^ FW)
Cd**^-^**	5.1 ± 0.17^b^	8.6 ± 0.57^b^	5.3 ± 0.10^b^	2.1 ± 0.30^b^	76.5 ± 7.4^b^
Cd^+^	1.7 ± 0.45^a^	4.5 ± 0.50^a^	3.6 ± 0.30^a^	1.2 ± 0.15^a^	40.2 ± 9.8^a^
Cd^+^ Si^+^	4.5 ± 0.70^b^	7.5 ± 0.50^b^	4.6 ± 0.65^b^	1.9 ± 0.15^b^	80.6 ± 14.3^b^
Cd^**-**^ Si^+^	1.9 ± 0.60^a^	6.6 ± 2.08a^b^	3.1 ± 0.64^a^	1.7 ± 0.56^ab^	79.4 ± 18.7^b^

*Different letters in each column indicate significant differences between mean ± SD of treatments (*n* = 3) at a *P* < 0.05 significance level.*

### Cd, Fe, and Si Contents in Roots and Shoots

**FIGURE 2:**
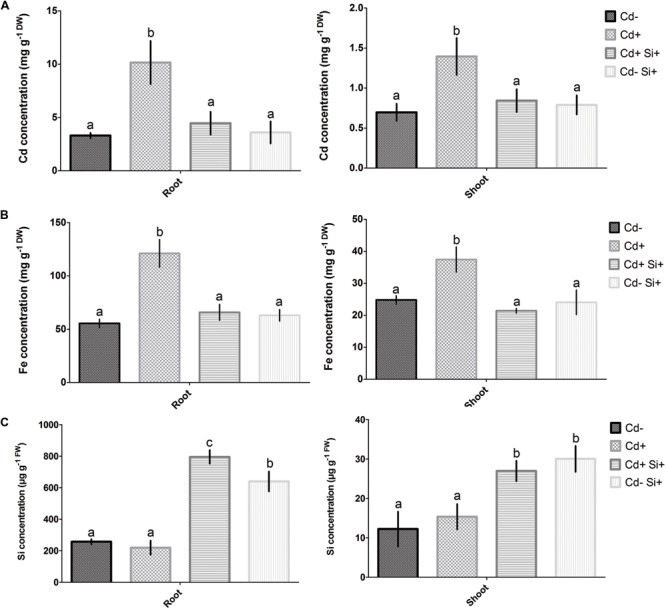
**Concentrations of Cd **(A)**, Fe **(B)**, and Si **(C)** in roots and shoots of 7-days-old Alfalfa plants grown under different growth conditions of Cd and Si.** Different letters in each column indicate significant differences between mean ± SD of treatments (*n* = 3) at a *P* < 0.05 significance level.

Cadmium and Fe contents increased in the whole plant under Cd stress compared with non-treated controls, showing the higher amount in roots than in shoots (**Figure 2A**). Supplementation with Si along with Cd significantly decreased the accumulation of Cd and Fe in both roots and shoots compared with the other three (i.e., Cd^-^, Cd^+^, Cd-Si^+^) treatments (**Figures 2A,B**). Plant showed lower Si concentration in roots when plants were grown without Si treatment compared with Si-treated plants grown with or without Cd. Further, Si concentration significantly increased in roots when plants were treated with Cd along with Si compared with the plants grown with or without Cd under hydroponic conditions (**Figure 2C**). Plants treated by Si without Cd showed significantly decreased Si concentration in roots compared to the plants grown under dual treatment of Si and Cd. In shoots, Si concentration considerably increased when plants were grown under Si supplementation with or without Cd compared to non-treated control and Cd-stressed plants (**Figure 2C**).

### Determination of Total Soluble Protein Content, Electrolyte Leakage, Fe Chelate Reductase Activity

**FIGURE 3:**
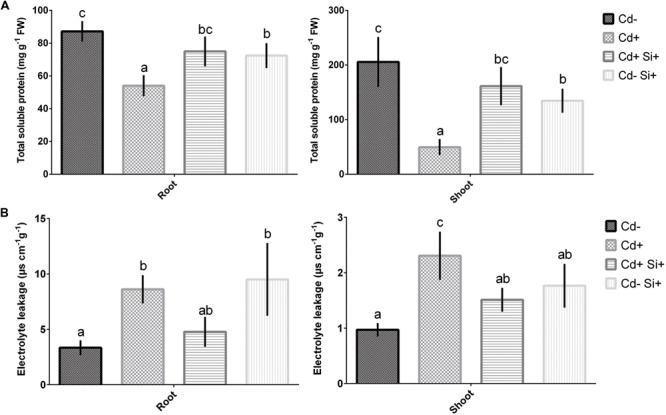
**Total protein content **(A)** and electrolyte leakage **(B)** in roots and shoots of Alfalfa plants grown under different growth conditions of Cd and Si.** Different letters in each column indicate significant differences between mean ± SD of treatments (*n* = 3) at a *P* < 0.05 significance level.

Cadmium stress caused a significant decrease in total protein content in both roots and leaves compared to control plants. However, application of Si along with Cd significantly increased the protein content in both tissues compared with Cd-stressed plants. When treated solely with Si on controls, plants showed protein content similar to that of the plants grown with Si and Cd supplementation (**Figure 3A**). The EL significantly increased in both roots and shoots under Cd stress compared with control plants (Cd^-^). However, the EL significantly decreased in both tissues due to Si along with Cd treatments compared with Cd-stressed Alfalfa (**Figure 3B**). Si applied solely in non-treated plants showed similar EL to that of plants treated with Cd and Si along with Cd in roots and shoots, respectively (**Figure 3B**).

**FIGURE 4:**
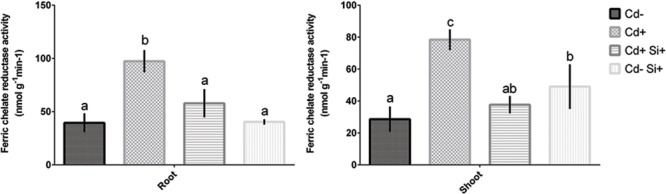
**Fe chelate reductase activity in roots and shoots of 7-days-old Alfalfa plants grown under different growth conditions of Cd and Si.** Different letters in each column indicate significant differences between mean ± SD of treatments (*n* = 3) at a *P* < 0.05 significance level.

Cadmium toxicity caused a significant increase in Fe-chelate reductase activity in both roots and shoots in comparison with control plants (**Figure 4**). However, Si supplementation along with Cd showed a significant decrease in Fe-chelate reductase activity compared with Cd-stressed plants (**Figure 4**). Si applied solely to non-treated plants showed similar Fe-chelate reductase activity in roots to that of controls and plants treated with Si and Cd. However, Fe-chelate reductase activity significantly increased in shoots compared to controls but showed similar activity to that of plants grown under both Si and Cd supplementations (**Figure 4**).

### HPLC Analysis in Roots

In this study, phytochelatin showed no significant changes in roots among the treatments in Alfalfa plants (**Table 2**). Further, citrate and malate concentrations significantly decreased due to Cd stress compared with non-treated controls. However, Si application to Cd-stressed plants showed a significant increase in citrate and malate concentrations in roots compared with the plants grown under Cd stress. Si applied solely to control plants showed similar citrate and malate levels to that of control plants (**Table 2**).

**Table 2:** Concentrations (μg**^-^**^1^ FW) of total phytochelatin, citrate and malate in roots of 7-days-old Alfalfa plants grown under different growth conditions of Cd and Si.

Treatments	Phytochelatin	Citrate	Malate
Cd**^-^**	1.54 ± 0.37^a^	1.33 ± 0.17^a^	1.24 ± 0.12^a^
Cd^+^	1.91 ± 0.70^a^	2.94 ± 0.73^b^	3.95 ± 0.47^b^
Cd^+^ Si^+^	1.86 ± 0.21^a^	1.06 ± 0.08^a^	0.85 ± 0.13^a^
Cd**^-^** Si^+^	1.91 ± 0.04^a^	0.94 ± 0.05^a^	1.99 ± 1.99^a^

*Different letters in each column indicate significant differences between mean ± SD of treatments (*n* = 3) at a *P* < 0.05 significance level.*

### Expression of Genes in Roots

**FIGURE 5:**
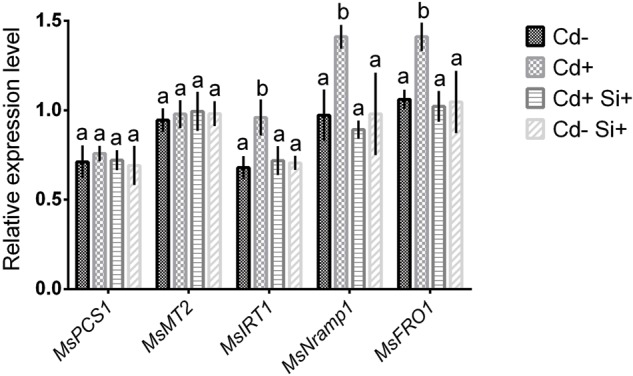
**Quantitative expression analysis of *MsPCS1, MsMT2, MsIRT1, MsNRAMP1, MsFRO1* transcripts in roots of Alfalfa plants grown under different growth conditions for 7-days.** Different letters in each column indicate significant differences between mean ± SD of treatments (*n* = 3) at a *P* < 0.05 significance level.

Quantitative real-time PCR analysis showed no significant changes in the expression of two metal chelators (*MsPCS1* and *MsMT2*) in roots of Alfalfa among the treatments (**Figure 5**). However, three Fe-related genes, *MsIRT1, MsNramp1*, and *MsFRO1* significantly upregulated in roots of Alfalfa plants under Cd stress compared with control plants. Further, expression of these genes significantly decreased when plants were treated with Si along with Cd (**Figure 5**). Si applied to non-treated plants showed similar expression pattern for these Fe-related genes to that of control plants (**Figure 5**).

### Enzymatic Changes in Roots

Enzymatic activity of CAT, APX, and SOD significantly decreased due to Cd stress compared with controls in roots of Alfalfa. However, application of Si along with Cd showed a significant increase in CAT, APX, and SOD activities compared with Cd-stressed plants (**Table 3**). Si applied solely to non-treated plants showed similar activities CAT and SOD to that of control plants. Further, POD and GR activities showed no significant changes in roots of Alfalfa among the treatments. Results also demonstrated that H_2_O_2_ content significantly increased due to Cd stress compared with controls in roots. However, application of Si with or without Cd showed a significant decrease in H_2_O_2_ content compared with Cd-stressed plants (**Table 3**).

**Table 3:** Enzymatic activities in roots of Alfalfa after cultivation for 7 days in nutrient medium with or without Cd and Si.

Treatments	CAT min^-1^ [(mg protein)^-1^]	APX min^-1^ [(mg protein)^-1^]	POD min^-1^ [(mg protein)^-1^]	SOD [U.mg^-1^ protein]	GR [nmol.NADH.min^-1^mg protein^-1^]	H_2_O_2_ content μmol g^-1^ FW
Cd**^-^**	1.07 ± 2.26^a^	3.46 ± 0.50^a^	0.20 ± 0.03^a^	0.19 ± 0.02^b^	0.51 ± 0.38^a^	0.33 ± 0.07^a^
Cd^+^	1.85 ± 0.46^b^	1.45 ± 0.32^b^	0.24 ± 0.05^a^	0.09 ± 0.03^a^	0.23 ± 0.10^a^	2.94 ± 0.79^b^
Cd^+^ Si^+^	3.08 ± 0.26^c^	3.82 ± 0.80^a^	0.20 ± 0.03^a^	0.19 ± 0.03^b^	0.16 ± 0.05^a^	1.10 ± 0.24^a^
Cd**^-^** Si^+^	0.61 ± 0.26^a^	3.94 ± 1.21^a^	0.21 ± 0.02^a^	0.13 ± 0.01^b^	0.23 ± 0.02^a^	0.78 ± 0.13^a^

*Different letters in each column indicate significant differences between mean ± SD of treatments (*n* = 3) at a *P* < 0.05 significance level.*

### Changes in Antioxidant Metabolites

We also studied the changes in some key metabolites having antioxidant properties. It was found that glutathione and cysteine showed no significant changes in roots of Alfalfa plants subjected to different treatments of Cd and Si (**Table 4**). However, Cd stress caused a significant decrease in methionine compared with controls but showed a significant increase when plants were treated with Cd and Si. Further, the investigation showed no significant reduction in proline concentration in response to Cd stress, application of Si along with Cd significantly increased proline concentration in roots of Alfalfa. Application of Si to control plants showed similar proline level to that of controls (**Table 4**).

**Table 4:** Concentrations (μg**^-^**^1^ FW) of antioxidant metabolites in roots of 7-days-old Alfalfa plants grown under different growth conditions of Cd and Si.

Treatments	Glutathione	Cysteine	Methionine	Proline
Cd**^-^**	9.8 ± 1.89^a^	5.6 ± 1.37^a^	2.7 ± 0.69^b^	4.1 ± 1.36^a^
Cd^+^	11.9 ± 2.42^a^	5.8 ± 0.70^a^	1.2 ± 0.25^a^	5.6 ± 2.8^a^
Cd^+^ Si^+^	11.3 ± 3.12^a^	5.1 ± 0.73^a^	6.8 ± 0.59^d^	12.3 ± 2.3^b^
Cd**^-^** Si^+^	12.0 ± 2.10^a^	5.7 ± 0.47^a^	4.9 ± 0.70^c^	6.8 ± 2.0^a^

*Different letters in each column indicate significant differences between mean ± SD of treatments (*n* = 3) at a *P* < 0.05 significance level.*

## Discussion

### Role of Si Restoring Morphological Features

In the present study, Si supplementation under Cd showed significant improvement in root and shoot growth parameters along with total chlorophyll content in Alfalfa. Also, application of Si in Cd-treated plants restored the total protein content in both roots and shoots compared with Cd-stressed plants. The role of Si increasing total soluble protein under drought stress is associated with the reduction of free radicals and ROS regeneration (Gong et al., 2005). Furthermore, application of Si in Cd-stressed plants diminished the EL in both roots and shoots. It indicates that exogenous Si might provide membrane stability in Cd-stressed Alfalfa plants. In a similar study, lead (Pb) stress induced a significant increase in the membrane injury but Si application noticeably eliminated EL in cotton plants (Bharwana et al., 2013). Although differences were not statistically significant, Si application under Cd stress did not completely restore morphological features back to that observed for untreated controls. This could be due to the concentration of Cd and Si used and can be optimized depending on the severity of stress involved. We also observed that Si was able to withstand the adverse effect of Cd on leaf expansion and stem elongation in Alfalfa. It might coincide with the role of silicon increasing soluble protein content and maintaining chlorophyll concentrations in shoots (Zhu et al., 2004; Farooq et al., 2013). Altogether, our findings support that Si can restore the damages and significantly improved the growth parameters provoked by Cd stress in Alfalfa var. Longdong plants due to its efficiency in utilizing exogenous Si. Genotypic variation in Cd accumulation and tolerance is reported in many plant species (Metwally et al., 2004; Yan et al., 2010; Pourghasemiana et al., 2013). Therefore, Si-mediated alleviation of Cd toxicity may behave differently in other cultivars of Alfalfa.

Atomic absorption spectroscopy data further showed that dual application of Si and Cd showed a reduction of Cd concentration to its normal level in both roots and shoots. It indicates that Si inhibits Cd uptake in roots and subsequently reduces the translocation of Cd into the shoots of Alfalfa plants. Similarly, Si-mediated reduction of Cd uptake was also reported in roots of cucumber (Wu et al., 2015). In the present study, we also observed higher accumulation of Fe in roots and shoots due to Cd stress. Interestingly, Si supplementation under Cd stress showed a significant decrease in Fe concentrations in both roots and shoots, being consistent with Cd concentration. These findings support that inhibition of Cd uptake in roots of Alfalfa plants is tightly regulated with Fe transport under Si supplementation. In the light of Si concentration in roots and shoots, it is evident that Alfalfa plants require more exogenous Si under Cd stress to withstand its toxicity. In this study, it was also noticed that exogenous Si applied to non-treated plants showed a slight reduction in root parameters. It may be possible that exogenous Si when not used by plants for Cd alleviation might hamper metabolism. Taken together, our findings indicate that alleviation of Cd toxicity in Alfalfa plants is largely dependent on the regulation of Cd and Fe uptake in root systems.

### Biochemical and Molecular Mechanisms Associated with Cd Detoxification

In the light of morpho-physiological observations, the mechanistic basis for Cd toxicity tolerance through Si supplementation was further investigated in roots of Alfalfa plants. We initially performed PC analysis and two candidate genes (*MsPCS1, MsMT2*) responsible for vacuolar sequestration in roots. HPLC analysis showed no significant changes in PC accumulation in roots under dual treatment of Si and Cd compared with control and Cd-stressed Alfalfa plants. Being consistent with PC accumulation, expression of *MsPCS1* and *MsMT2* showed no significant changes in roots of Alfalfa due to Si effect. Induction of PC and MT is involved in vacuolar sequestration of heavy metal is plants. However, Greger et al. (2016) suggested that the inhibition of Cd uptake in wheat closely associated with the increased Cd extrusion from the cells than PC accumulation. Our studies indicate that Cd detoxification in Alfalfa plants following Si application may not be associated with the change in metal sequestrations in roots. This observation is correlated with AAS data revealing no significant Cd accumulation in roots due to Si application. Therefore, it might be possible that effect of Si on Cd-stressed Alfalfa is dependent on the inhibition of metal transporters in root systems.

As there are no specific Cd transporters in plants, uptake of Cd is achieved by transporter genes located on plasma membranes (Morel et al., 2009). To characterize the Fe-chelating potential under Si supplementations in Alfalfa, we further studied the changes of Fe related genes (*MsIRT1, OsNRAMP1*, and *MsFRO1*) along with Fe-reductase activity in roots. Expression of these genes and Fe-reductase activity significantly decreased due to Si application under Cd stress in Alfalfa plants, indicating that regulation of Fe uptake and chelation plays a critical role in limiting Fe-mediated Cd accumulation in Alfalfa plants under Si supplementation. Fe-chelate reductase activity is known as an important biochemical mechanism regulating Fe availability in plant cells. FROs genes may be required for Fe distribution within aerial portions of the plant and/or may be involved in Fe transport mechanisms in the organelle (Jeong and Connolly, 2009). Changes of transcription factors belonging to several families demonstrate that responses of plants under Cd stress are very complex (Fusco et al., 2005; Weber et al., 2006). Transcription factors regulating genes involved in Cd detoxification following Si supplementation might provide interesting message to future researchers. Our data further showed a significant increase in two Fe chelators (citrate and malate) in roots due to Cd stress revealing that the chelation of Fe through the organic acids is associated with enhanced Fe as well as Cd uptake in Alfalfa. Interestingly, Si application to Cd-stressed plants resulted in a decrease in both citrate and malate concentrations. It indicates that Si might indirectly decrease Cd accumulation through the inhibition of Fe chelation strategies in Alfalfa plants.

### Antioxidant Defense under Cd Stress

We also analyzed antioxidant enzymes involved in antioxidant defense in plants under abiotic stress. Among these enzymes, CAT and SOD activities significantly increased in roots under Si supplementation in Cd-stressed Alfalfa plants. CAT is involved in the main defense mechanism against accumulation and toxicity of ROS and may play a fundamental role in decreasing H_2_O_2_ level in plant cells (Malar et al., 2014). SOD provides a front line defense under the metal-induced oxidative damage and catalyzes the conversion of the superoxide radical to molecular oxygen and H_2_O_2_ (Romero-Puertas et al., 2007; Irfan et al., 2014). In the present work, Alfalfa plants have a tendency to increase SOD activity under Cd stress, suggesting better efficiency in converting O_2_ to H_2_O_2_ following Si treatment. The higher CAT and SOD activities due to Si on Cd-stressed Alfalfa plants may be partially correlated with the 0decreased H_2_O_2_ concentrations in roots. CAT mainly occurs in peroxisomes and does not require a reductant for catalyzing a dismutation reaction (Sofo et al., 2015). Wu et al. (2015) also reported that the Si-mediated increases in antioxidant enzymes might be an adaptive response to counteract Cd stress in tomato. However, the increase of APX due to Si supplementation under Cd stress indicates that inhibition of H_2_O_2_ might be associated with its increased activity. APX is largely intracellular and involved in the control of cellular H_2_O_2_ levels in chloroplasts, cytosol, mitochondria and peroxisomes, and apoplastic space (Veitch, 2004; Sofo et al., 2015). Further, APX activity increases along with other enzymes activities, such as CAT, SOD, and GSH reductase (Shigeoka et al., 2002). In another study, Cd and Cu exposures caused the increase in APX and SOD activities (Lee et al., 2007). These results emphasize that Si-mediated scavenging of Cd-induced ROS might be correlated with APX and SOD but not POD in Alfalfa plants.

Our studies also revealed the increase of methionine and proline due to Si treatment on Cd-stressed plants. Methionine can undergo ROS-mediated oxidation to methionine sulfoxide by methionine sulfoxide reductases to ameliorate oxidative damage (Dos Santos et al., 2005; Cabreiro et al., 2006). Also, methionine is a substrate for the synthesis of various polyamines (putrescine, spermidine, spermine) associated with stress tolerance (Alcázar et al., 2010) and methionine residues act as an antioxidant protein reservoir (Hoshi and Heinemann, 2001). Apart from acting as an osmolyte, proline scavenges free radicals, stabilizes sub-cellular structures and proteins under stress conditions (Sharma and Dubey, 2005). Thus, our results suggest that induction of antioxidative metabolites help Alfalfa seedlings to adapt better to the Cd stress. Metal-induced proline accumulation in plants is not directly originated from heavy metal stress, but for water balance disorder by regulating the contents of inorganic solutes (Rai, 2002; Clemens, 2006). Furthermore, proline synthesis is advantageous to NADP^+^ refilling and redox cycling, which is of particular importance for antioxidant defense mechanisms in plants during stress (Babiychuk et al., 1995). In this study, Si did not affect the concentration of glutathione and cysteine, which is correlated with the PC accumulation in roots as these two metabolites are involved in PC biosynthesis pathway. Therefore, it turned out that the active involvement of ROS scavenging mainly mediated by elevated SOD, methionine and proline was related, at least in part, to the Si-mediated alleviation of Cd stress in Alfalfa.

## Conclusion

In this present study, morpho-physiological findings support that Si plays a critical role to withstand Cd toxicity and to restore the normal growth and development in Alfalfa plants. Further, simultaneous reduction of Cd and Fe in roots and shoots of Alfalfa due to Si treatment under Cd stress indicates that mechanisms counteracting Cd toxicity exist in roots. Further, no changes of metal chelator (*MsPCS1* and *MsMT2*) genes and PC synthesis in roots suggest that changes in metal sequestration may not be involved in Si-mediated mitigation of Cd toxicity in Alfalfa. Also, reduction of Fe-reductase activity, downregulation of Fe-related genes (*MsIRT1, MsNramp1*, and *MsFRO1*) and a decrease of Fe chelators (citrate and malate) reveals that alleviation of Cd toxicity in Alfalfa plants largely depends on the inhibition of Fe uptake and Fe chelation in roots. Finally, a Si-mediated increase in antioxidant enzymes (CAT, SOD, APX) and metabolites (methionine, proline) might play an integral part against Cd-induced oxidative stress in Alfalfa plants. These findings may stimulate further researches for phytoremediation and development of Cd-tolerant crops through Si treatment.

## Author Contributions

AK conduced most of the laboratory works and prepared the draft manuscript. MH and MK performed few laboratory works. AM and SH revised the manuscript.

## Conflict of Interest Statement

The authors declare that the research was conducted in the absence of any commercial or financial relationships that could be construed as a potential conflict of interest.
