# Supplementary material for: Role of Silicon Counteracting Cadmium Toxicity in Alfalfa (Medicago sativa L.)
Source: Front Plant Sci. 2016 Jul 27;7:1117. doi: 10.3389/fpls.2016.01117 (PMC4961700; doi:10.3389/fpls.2016.01117)
Supplement: Supplementary file 1 [file Table_1.DOCX]

**Supplementary Table S1.** List of primers used for qPCR.

| Gene Name | Primer sequences |
| --- | --- |
| *Actin* | Forward: ACGAGCGTTTCAGATG  Reverse: ACCTCCGATCCAGACA |
| *MsPCS1* | Forward: AGGTATTGGGACAGGTGCAG  Reverse: ATGCTGCCGATGAGCTAAGT |
| *MsMT2* | Forward: CATAATGAAATGAAACTA  Reverse: CGGGGACAGATCATCA3 |
| *MsIRT1* | Forward: AGTGCTCGTCCAAATATGAAGGTG  Reverse: TGCTGGGATCGAAGTTGTGAAA |
| *MsNramp1* | Forward: AAGGGGTTTGTAAACGATGGTCAGT  Reverse: GCTCTCTAAAATGACTTGAAAGCAA |
| *MsFRO1* | Forward: GGTGACACGTGGATCATCTG  Reverse: TTGCAATCCACAGGAACAAA |
